# Manuka Honey Modulates the Inflammatory Behavior of a dHL-60 Neutrophil Model under the Cytotoxic Limit

**DOI:** 10.1155/2019/6132581

**Published:** 2019-02-25

**Authors:** Benjamin A. Minden-Birkenmaier, Kasyap Cherukuri, Richard A. Smith, Marko Z. Radic, Gary L. Bowlin

**Affiliations:** ^1^Department of Biomedical Engineering, University of Memphis, 3806 Norriswood Ave., Memphis, TN 38152, USA; ^2^Department of Orthopaedic Surgery & Biomedical Engineering, University of Tennessee Health Science Center, 956 Court Ave., Memphis, TN 38163, USA; ^3^Department of Microbiology, Immunology and Biochemistry, University of Tennessee Health Science Center, 858 Madison Ave., Memphis, TN 38163, USA

## Abstract

Recent work has shown that Manuka honey, an increasingly popular wound additive with potent antibacterial properties, also has anti-inflammatory properties. However, little research has been done examining its effect on neutrophils. This study investigates the hypothesis that Manuka honey reduces neutrophil superoxide release and chemotaxis and reduces the activation of the inflammatory nuclear factor-*κ*B (NF-*κ*B) signaling pathway under honey's cytotoxic limit. A differentiated HL-60 cell line was used as a neutrophil model and cultured in various concentrations of Manuka honey for 3 and 24 hours to measure cytotoxicity via mitochondrial activity and visual trypan-exclusion count. Cytochrome C and Boyden chamber assays were used to measure the effect of Manuka honey on superoxide release and chemotaxis toward fMLP, respectively. Additionally, a Western blot for NF-*κ*B inhibitor *α* (I*κ*B*α*) was performed to measure Manuka honey's effect on the NF-*κ*B pathway via I*κ*B*α* phosphorylation. The results indicate a cytotoxic limit of 3-5% v/v. The presence of 1% honey decreased superoxide release at 24 hours. The 0.5, 1, and 3% honey concentrations reduced chemotaxis and I*κ*B*α* phosphorylation in a dose-dependent fashion. These results suggest that Manuka honey significantly reduces neutrophil recruitment and inflammatory behavior in the wound site in a dose-dependent fashion under the cytotoxic limit.

## 1. Introduction

Studies have demonstrated that topical application of honey to wounds promotes wound closure, induces an osmotic gradient which cleanses the wound via fluid movement, reduces wound inflammation, and inhibits the growth of a range of bacteria varieties [1–4]. The high concentration of sugars in the honey creates an osmotic gradient that pulls fluid from the subcutaneous tissue up through the wound area, flushing necrotic debris from the wound site and carrying nutrients and oxygen from the surrounding area into the damaged tissue [5]. Additionally, this gradient helps to remove excess fluid from the wound environment, which has been shown to impede bacterial growth [6]. Flavonoids within the honey scavenge free oxygen radicals, reducing inflammation and minimizing tissue damage [7–9]. Previous work by Alvarez-Suarez* et al.* has analyzed the phenolic content of Manuka honey via HPLC-MS, and it is theorized that these components improve the intracellular antioxidant response [10]. In addition, honey's hydrogen peroxide content acts as an antiseptic against many types of bacteria [11–13]. These properties and others have been reviewed in detail in previously published literature [5, 14–20].

The anti-inflammation and prohealing properties of Manuka honey have led some groups to incorporate it as an additive within biomaterials such as tissue engineering templates [21–25]. As the implantation of these templates requires the creation of a wound site and the associated increase in neutrophil presence, the effect of Manuka honey on neutrophil activity is relevant to this line of research. Excessive neutrophil inflammatory activity has been implicated in the initiation of fibrosis, which can impede tissue-biomaterial integration [26]. The ability of Manuka honey to modulate such neutrophil inflammatory activity would increase its usefulness as a template additive. Of particular importance to this research are potential cytotoxic effects of the honey, which could inhibit cell infiltration and proliferation within these templates. As such, it is necessary to determine the concentration at which honey becomes cytotoxic to neutrophils and to investigate a range of honey concentrations to determine the optimum loading and release levels for tissue engineering templates.

In this study, a specific variety of honey termed Manuka honey is used. In addition to the effects described above, Manuka honey contains a methylglyoxal component which imbues it with additional antimicrobial activity [4, 27]. This methylglyoxal component is primarily responsible for the Unique Manuka Factor (UMF), a term used by the industry to describe the heightened antimicrobial activity of Manuka honey. After Manuka honey is collected, it is subject to a bacterial inhibition test, and the UMF is defined as the concentration of phenol necessary to achieve the bacterial inhibition of that Manuka honey sample (for instance, Manuka honey with a UMF of 15 would exhibit the same bacterial inhibition as 15% phenol) [28]. This test is standardized and used across the industry to compare the antimicrobial effects of various Manuka honey products [23, 29, 30]. As the properties of Manuka honey can vary slightly based on area of collection and processing parameters, this test allows for a standardized measurement of the honey's bacterial inhibition properties.

Although the role of neutrophils has been classically considered to be primarily phagocytic, recent research has demonstrated the ability of these cells to regulate wound healing through the release of growth factors, chemo/cytokines, and proteases [31]. These cells arrive through the bloodstream, travel via chemotaxis to the wound site soon after the occurrence of injury, and begin fighting bacterial invasion via phagocytosis, superoxide release, and the extrusion of neutrophil extracellular traps (NETosis) [32]. Additionally, they release a wide variety of factors that recruit more neutrophils, macrophages, and other inflammatory cells and also amplify the overall inflammatory response [33]. While this proinflammatory neutrophil activity is effective at fighting bacterial invasion, the factors released by these cells can damage native tissue, impairing wound healing [34]. However, in addition to this proinflammatory behavior, neutrophils also have the ability to exhibit anti-inflammatory, proresolution behaviors [35, 36]. These behaviors include inhibiting additional neutrophil recruitment to the wound site, downregulating the degranulation of mast cells, releasing anti-inflammatory IL-10, promoting angiogenesis, and inhibiting T-cell activation [34, 35, 37, 38]. The balance of these neutrophil behaviors is important in both promoting acute inflammation and transitioning the wound from inflammation to resolution and healing, and thus avoiding chronic inflammation [32, 35]. Given the importance of neutrophils in controlling wound inflammation, as well as the growing clinical use of Manuka honey as a wound additive with demonstrated anti-inflammatory properties, the effect of Manuka honey on neutrophil behavior is of scientific interest [39].

One important indicator of the activation of the inflammation response of the neutrophil is the phosphorylation of I*κ*B*α*, a regulatory protein which inhibits the inflammatory nuclear transcription factor NF-*κ*B by trapping it in the cytoplasm. NF-*κ*B is a transducer for many inflammatory pathways [40–43]. These signal cascades trigger the phosphorylation of I*κ*B*α*, which is bound to NF-*κ*B. As I*κ*B*α* is phosphorylated, it releases the NF-*κ*B molecules, allowing them to enter the nucleus and bind to promoter regions in the DNA. By binding to these regions, NF-*κ*B upregulates the transcription of inflammation response genes [44–46].

In this study, the human leukemia HL-60 cell line was differentiated and used as a model of the neutrophil. This neutrophil model has been extensively characterized [47–49]. Certain behaviors of primary neutrophils can vary substantially from donor to donor, including substrate adherence, chemotaxis, cytokine release, and damage to surrounding cells/tissues [50–52]. Utilizing the dHL-60 model eliminates this variability and provides a reliable standard that can be used by others in the field. These differentiated cells were polarized with lipopolysaccharide (LPS) and formylmethionine-leucyl-phenylalanine (fMLP) as inflammatory stimuli or transforming growth factor *β*1 (TGF-*β*) as an anti-inflammatory stimulus. These cells were cultured in a range of concentrations of Manuka honey, and their behavior was characterized with regard to cytotoxicity, superoxide release, chemotaxis, and I*κ*B*α* phosphorylation.

The hypothesis of this study is that Manuka honey reduces neutrophil inflammatory behavior, specifically superoxide release, chemotaxis to the bacterial signal fMLP, and I*κ*B*α* phosphorylation, when present under the cytotoxic limit.

## 2. Materials and Methods

### 2.1. HL-60 Culture and Differentiation

HL-60s were purchased from the American Type Culture Collection (ATCC, CCL 240, Manassas, VA, USA) and cultured at a cell density range of 2 x 10^5^ to 1 x 10^6^ cells per mL in culture media consisting of RPMI (Hyclone, Logan, UT, USA) with 10% v/v non-heat-inactivated fetal bovine serum (FBS) (Hyclone), 1% v/v penicillin/streptomycin (Pen/Strep) (Corning, NY, USA), and 1% L-glutamine (Gibco, Billings, MT, USA) (referred to as culture medium). Cells were incubated at 37°C in a 5% CO_2_ environment in T-25 and T-75 culture flasks (Thermo Scientific, Rochester, NY, USA). Medium was changed every 3-4 days and cells were passaged when cell density reached 5 x 10^5^ cells/mL. Cells were used for passage numbers up to 30. These cells were differentiated to a neutrophil-like phenotype by adding 1.25% dimethylsulfoxide (DMSO) (Fisher Scientific, Hampton, NH, USA) to the culture medium for six days, replenishing the medium/DMSO on the third day. This procedure has been validated in previous studies [27, 28].

### 2.2. Confirmation of Differentiation

Differentiation was confirmed morphologically by permeabilizing with 0.17 mM Triton X-100 (Fisher Scientific) for 5 minutes, then fixing in 4% buffered paraformaldehyde (Fisher Scientific) and staining with 4',6-diamidino-2-phenylindole (DAPI) (NucBlue Fixed Cell Stain ReadyProbes reagent) for 5 minutes at stock concentration and phalloidin-conjugated Alexa Fluor 488 (ActinGreen 488 ReadyProbes reagent) (both from Invitrogen, Carlsbad, CA, USA) for 30 minutes also at stock concentration according to the manufacturer's protocols. Cells were imaged with an Olympus microscope (model BX34F) with an attached Olympus DP73 digital color camera and Olympus U-HGLGPS fluorescent light source (Olympus, Shinjuku, Tokyo, Japan). The percentage of differentiated cells (kidney-shaped nucleus) was calculated to be 69%, comparable to the percentage reported in literature (see Supplemental Section 1) [47, 53].

### 2.3. Cell Stimulation

Differentiated HL-60s (dHL-60s) were incubated in the presence of inflammatory and anti-inflammatory stimuli. Proinflammatory responses were elicited by incubating dHL-60s with 1 *μ*g/mL LPS (Invivogen, San Diego, CA, USA) for 90 minutes, then adding 10^−7^ M fMLP (Sigma Aldrich, St. Louis, MO, USA) immediately prior to use. Work by Nath et al. has previously shown these concentrations of LPS and fMLP with a 90-minute polarization step to effectively stimulate superoxide release in neutrophils [54]. Anti-inflammatory responses were elicited by incubating dHL-60s with 2 ng/mL TGF-*β*1 (Gibco) for 24 hours prior to use. While TGF-*β* levels on the order of pg/mL have been shown to elicit neutrophil migration, levels on the order of 2 ng/mL have been shown to exist within the healing wound environment during peak neutrophil recruitment [55, 56]. Unstimulated dHL-60s were used as an additional experimental control.

### 2.4. Mitochondrial Activity Assay

To begin this assay, 400,000 of non-stimulated (NS), inflammatory stimulated (LPS + fMLP), and anti-inflammatory stimulated (TGF-*β*) dHL-60s were seeded in 150 *μ*L culture media in a 96-well plate in 0, 0.1, 0.5, 1, 3, 5, 10, and 20% v/v concentrations of Manuka honey/culture medium (UMF 12+, Manuka Guard, Monterey, CA, USA, density of 1.51 g/mL) alongside honey/medium blanks containing no cells. At 0, 3, and 24 hours, 30 *μ*L of MTS solution (CellTiter 96 Aqueous One Solution Cell Proliferation Assay, Promega, Madison, WI, USA) was added to each well including the honey/medium blanks. After incubation at 37°C for 1 hour, the absorbances of the samples were read at 490 nm using a SpectraMax i3x Multi-Mode Detection plate reader (Molecular Devices, Sunnyvale, CA, USA). The absorbances of the honey/medium blanks were subtracted from the corresponding samples, and the results were expressed as relative mitochondrial activity. Statistical significance was measured via a two-way analysis of variance (ANOVA) with a Holm-Sidak post hoc (*α*=0.05).

### 2.5. Trypan-Exclusion Assay

To conduct this assay, 400,000 of NS, LPS + fMLP-stimulated, and TGF-*β*-stimulated dHL-60s were seeded in 150 *μ*L in a 96-well plate in 0, 0.1, 0.5, 1, 3, 5, 10, and 20% v/v of Manuka honey/medium for 3 and 24 hours. After culturing for their respective time periods, the liquid and cells from each well were pipetted into microcentrifuge tubes. Then, 30 *μ*L of trypsin (Gibco) was pipetted into each well and incubated for 5 minutes at 37°C to remove any remaining adherent cells. After the incubation period, the trypsin was pipetted out of each well and added to the corresponding microcentrifuge tube, which was centrifuged at 200 X G for 10 minutes. Supernatants were discarded, and cells were resuspended in 75 *μ*L culture media with 75 *μ*L trypan blue (Gibco). The numbers of viable and nonviable cells in each sample were counted with a Countess II FL Automated Cell Counter (Invitrogen). A two-way ANOVA with a Holm-Sidak post hoc was performed to test for statistical significance between groups (*α*=0.05).

### 2.6. Superoxide Production Assay

For this study, 150,000 NS, LPS + fMLP-stimulated, and TGF-*β*-stimulated dHL-60s were seeded in 150 *μ*L culture media in 96-well plates in 0, 0.1, 0.5, 1, 3, 5, 10, and 20% v/v of honey with 100 *μ*M ferricytochrome C (Sigma Aldrich) in accordance with a previously defined procedure [54, 57, 58]. After 1, 3, and 24 hours of culture, the absorbance at 550 nm was measured using the Spectramax plate reader. Honey/medium blanks of each honey concentration were run alongside the cell samples, and the absorbances of these blanks were subtracted from the cell samples to get the absorbance due to ferricytochrome C reduction. The results were displayed as relative superoxide production. Statistical significance was measured via a two-way ANOVA with a Holm-Sidak post hoc (*α*=0.05).

### 2.7. Chemotaxis Assay

Polystyrene transwell inserts with a 6.5-mm diameter polyester membrane perforated by 3.0 *μ*m diameter pores (Costar, Kennebunk, ME, USA) were used to measure chemotaxis via an adaptation of a previously defined method [59]. Briefly, 500,000 NS dHL-60s were seeded in the top inserts in 100 *μ*L of culture medium, and 650 *μ*L of culture medium with 50 nM fMLP and 0, 0.5, 3, and 20% honey was placed in the bottom chamber (fluid levels were the same height when the top inserts were placed onto the bottom chambers to avoid net fluid flow from one chamber to another). One additional control was run of NS cells seeded in top inserts above chambers containing medium with no honey or fMLP to establish the amount of cell movement that happens in the absence of a chemokine. Samples were incubated for 3 hours. At the end of the incubation period, the top inserts were removed from the bottom chambers. The contents of each top and bottom chamber were pipetted out into microcentrifuge tubes. 30 *μ*L of trypsin was added to each top chamber, and 650 *μ*L of trypsin was added to each bottom chamber, and then the plates were incubated at 37°C for 5 minutes. Trypsin was then removed from each top and bottom chamber and added to the respective microcentrifuge tubes, which were then centrifuged at 200 x G for 10 minutes. Supernatants were removed and discarded, and each sample was resuspended in 75 *μ*L of RPMI and 75 *μ*L of trypan blue. The numbers of viable and nonviable cells in each sample were then counted with a Countess II FL Automated Cell Counter (Invitrogen). Statistical significance was measured via a two-way ANOVA with a Holm-Sidak post hoc (*α*=0.05).

### 2.8. IKB*α* Phosphorylation Western Blot

Preliminary experimentation indicated that peak IKB*α* phosphorylation occurs 38 minutes after the addition of LPS and fMLP at the same time to the culture medium (results shown in Supplemental section 2). Accordingly, in this set of experiments the LPS was added with the fMLP at time 0, rather than 90 minutes before time 0 as was done in the above experiments. TGF-*β* was still added to its group 24 hours before time 0. NS, LPS + fMLP, and TGF-*β*-stimulated dHL-60s were seeded at 400,000 cells per well in 150 *μ*L of culture media in 96-well plates with 0, 0.5, and 3% v/v Manuka honey and incubated for 38 minutes. Plates were then placed on ice and cells were removed and lysed in 50 *μ*L radioimmunoprecipitation buffer (RIPA buffer, Thermo Scientific), using trypsin to remove remaining cells. Lysates were centrifuged at 13,000 x G at 4°C for 15 minutes to pellet the cell membrane detritus, and the supernatant was saved. Samples were denatured with lithium dodecyl sulfate (LDS) sample buffer and dithiothreitol (DTT) reducing agent (both from Invitrogen) at 70°C for 10 minutes. Proteins were subjected to gel electrophoresis using 3-8% Tris-Acetate gels (Invitrogen) and transferred to polyvinylidene difluoride membranes (Millipore, Burlington, MA, USA). Membranes were washed 5 times with Tris-buffered saline (TBS, Thermo Scientific), blocked with TBS blocking buffer (Thermo Scientific) for an hour, and then incubated overnight at 4°C in mouse anti-human IKB*α* primary antibody (MAB4299, R&D Systems, Minneapolis, MN, USA) at a 0.1 *μ*g/mL concentration and rabbit anti-human phospho-IKB*α* (S32/S36) primary antibody (AF4809, R&D Systems) at a 1 *μ*g/mL concentration in TBS blocking buffer. Following 5 washes in TBS with 0.1% Tween 20 (Fisher Scientific), the membrane was incubated at room temperature in IRDye 680RD Donkey anti-mouse secondary (P/N 925-68072) and IRDye 800CW Donkey anti-rabbit secondary (P/N 925-32213) (both from Li-Cor Biosciences, Lincoln, NE, USA) at a 1:20,000 dilution in TBS blocking buffer with 0.1% Tween 20 and 0.01% sodium dodecyl sulfate (Fisher Scientific) for one hour. The membrane was washed 3 times in TBS with 0.1% Tween 20, one time in TBS, and then scanned on an Odyssey CLx infrared imaging system (Li-Cor Biosciences). The relative fluorescence of the 800 nm and 700 nm channels was calculated for the relevant bands of each sample, subtracting out background fluorescence from the area around the bands using Image Studio™ version 5.2 software. Samples were run in groups of 3 concurrent lanes on two separate Western blots (total of 6 samples per group). Each Western blot had 3 lanes of non-stimulated dHL-60s cultured without honey, and the relative fluorescence ratio of all other samples was normalized to this control. Statistical significance was measured via a two-way ANOVA with a Holm-Sidak post hoc (*α*=0.05).

## 3. Results

### 3.1. MTS Mitochondrial Assay

**Figure 1:**
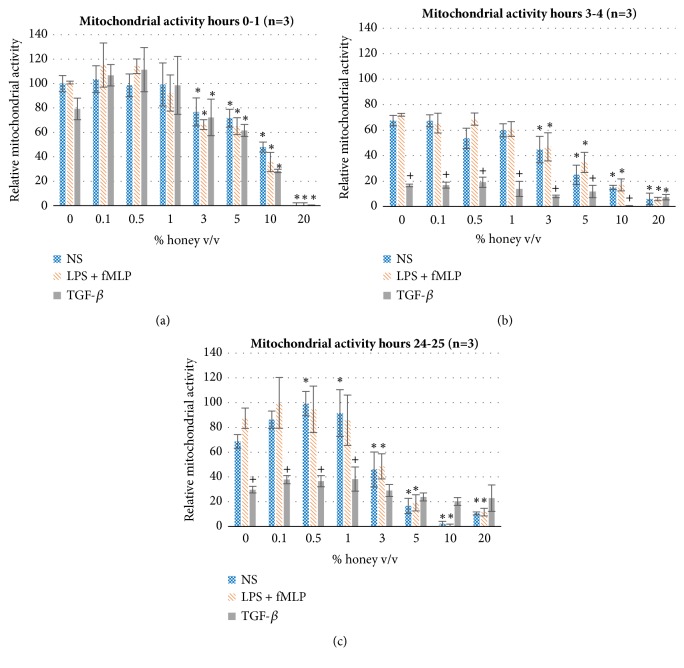
Mitochondrial activity of NS, LPS/fMLP, and TGF-*β*-stimulated dHL-60s at various concentrations of honey for hours (a) 0-1, (b) 3-4, and (c) 24-25 of culture. Values are normalized relative to the 0-1-hour NS 0% honey mitochondrial activity. *∗* indicates statistical significance from the respective 0% honey value of that phenotype/timepoint, and + indicates statistical significance from the other two phenotypes at that honey concentration and timepoint. *α*=0.05, measured via two-way ANOVA with Holm-Sidak post hoc.

Figure 1 shows the mitochondrial activity of cells of all three stimulation groups incubated at various concentrations of honey in (a) the first hour of incubation, (b) from hours 3 to 4, and (c) from hours 24 to 25. as shown by the black asterisks, a statistically significant decrease in mitochondrial activity begins at 3% honey and becomes more pronounced as honey concentration increases. This effect becomes stronger at the 3-4-hour and 24-25-hour time windows, suggesting a cytotoxic effect of the honey that begins in the range of 3-5% and becomes more accentuated at higher honey concentrations. These data sets also show that the mitochondrial activity of the TGF-*β*-stimulated cells decreases relative to the other two groups at the 3-4-hour and 24-25-hour time windows. This finding could indicate that the anti-inflammatory effect of the TGF-*β* stimulation decreases the overall cellular metabolism, although further studies would be necessary to confirm this hypothesis. As shown in Figure 1(c), the mitochondrial activity of the NS group is significantly upregulated at 0.5% and 1% honey, possibly indicating increased cellular metabolism of this phenotype at these intermediate honey concentrations.

### 3.2. Trypan Exclusion Assay

**Figure 2:**
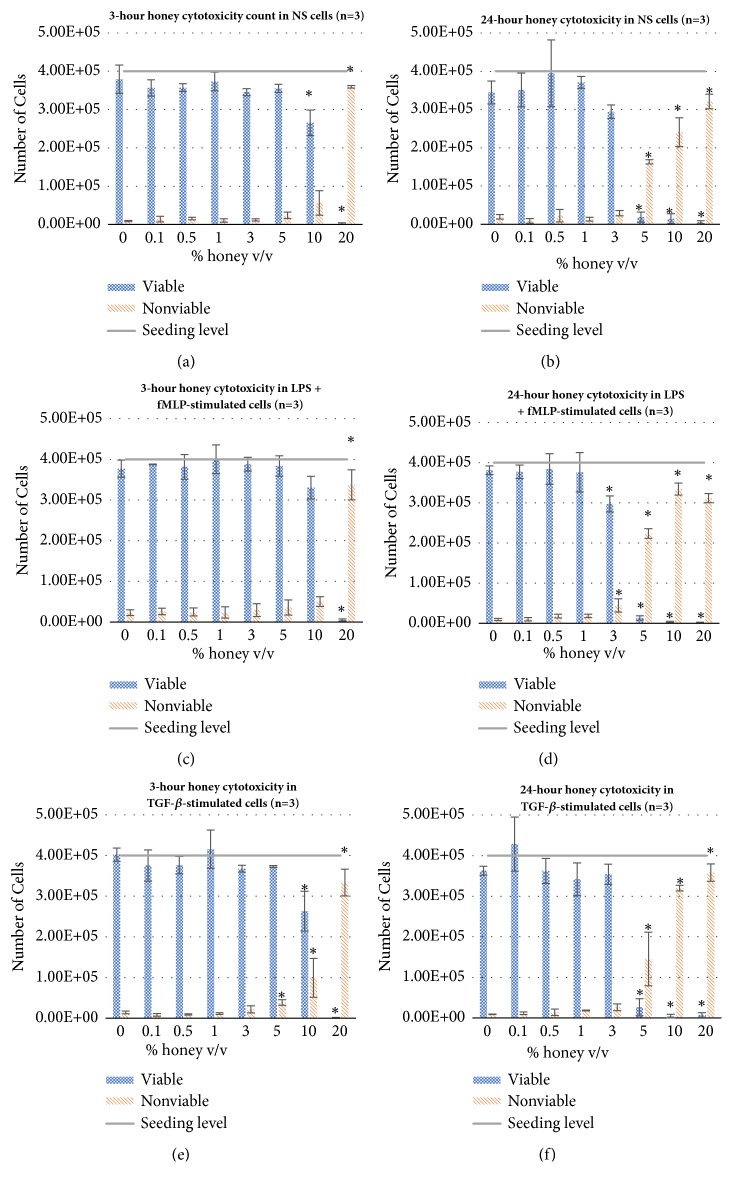
Viable and nonviable cell counts of NS, LPS + fMLP-stimulated, and TGF-*β*-stimulated dHL-60s cultured at each honey concentration for 3 (a, c, e) and 24 (b, d, f) hours. *∗* indicates a statically significant difference from the respective 0% honey control of each cell type, viable or nonviable, at each timepoint. *α*=0.05, measured via one-way ANOVA with Holm-Sidak post hoc.

Figure 2 displays the number of viable (trypan-excluding) and nonviable (non-trypan-excluding) cells of each stimulation group after 3 and 24 hours of culture with various concentrations of honey. At the 3-hour timepoint, there is a significant decrease in viable (a) NS, (c) LPS + fMLP, and (e) TGF-*β*-stimulated cells and an increase in nonviable cells of each group at 20% honey, with this trend beginning at 5% honey in the TGF-*β* group and 10% honey in NS group, indicating cytotoxicity at these concentrations. At the 24-hour timepoint, this significant dropoff in viable (d) LPS + fMLP-stimulated cells and increase in nonviable cells are seen at 3% honey and up, and in (b) NS and (f) TGF-*β*-stimulated cells, this effect happens at 5% honey and up. These data confirm the findings from the mitochondrial activity data that there is weak cytotoxicity in the 3-5% honey range and strong cytotoxicity at concentrations higher than 5% honey. The counts of the TGF-*β* group at 3 and 24 hours also confirm that the drop in mitochondrial activity that occurred in this group at 3-4 hours and 24-25 hours is not a result of increased cell death of this phenotype, but rather decreased mitochondrial activity of viable cells relative to the other two phenotypes.

### 3.3. Superoxide Production

**Figure 3:**
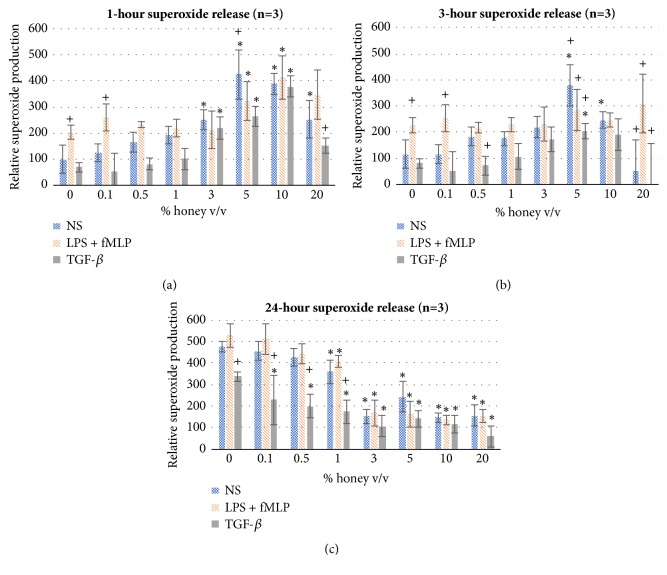
Superoxide production of the NS, LPS + fMLP-stimulated, and TGF-*β*-stimulated dHL-60s cultured at each honey concentration for (a) 1, (b) 3, and (c) 24 hours. Values are shown relative to the 0% honey NS 1-hour value. *∗* indicates statistical significance from the respective 0% honey value of that phenotype, and + indicates statistical significance from the other two phenotypes. *α*=0.05, measured via two-way ANOVA with Holm-Sidak post hoc.

Figure 3 shows the superoxide production of all three stimuli groups after 1, 3, and 24 hours of culture. As expected, the LPS + fMLP samples have significantly greater superoxide production at 0% honey at 1 and 3 hours relative to the other two stimuli groups (positive control). However, this difference decreases as honey concentration increases and superoxide production increases in the NS and TGF-*β* groups. This trend indicates that, within the first three hours, concentrations of 3% honey and above stimulate superoxide production in equal to or in excess of the superoxide production stimulated by LPS and fMLP in both NS and TGF-*β*-stimulated neutrophils and also further stimulate superoxide production in the LPS + fMLP group. However, as shown in Figure 3(c) at the 24-hour timepoint, there is an opposite trend with regard to honey concentration. At levels of 3% honey and above, superoxide levels significantly decrease in all three groups relative to the 0% honey control. Part of this decrease is likely due to the cytotoxic effect of these higher honey concentrations (addressed in the discussion section). Additionally, the TGF-*β* group has lower superoxide production than the other groups at intermediate honey concentrations (0.5% and 1%) at 1 and 3 hours (significant at 0.5% honey at the 3-hour timepoint) and has significantly less superoxide production relative to the other two phenotypes at the 24-hour timepoint at honey concentrations of 1% and lower. However, these cells did significantly increase their superoxide production at 3%, 5%, and 10% honey at the 1-hour mark and at 5% honey at the 3-hour timepoint, relative to their 0% honey control. At the 24-hour timepoint, the total cell numbers (trypan-excluding and not trypan-excluding) at 5% honey are lower than the seeded number. This discrepancy could be due to the destruction of nonviable cells or the phagocytosis of the nonviable cells by the live ones. However, additional experimentation would be required to confirm this assumption.

### 3.4. Chemotaxis

**Figure 4:**
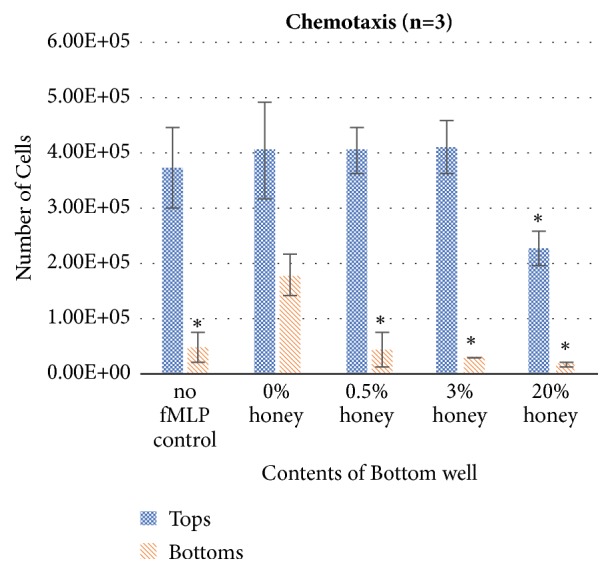
Chemotaxis of 500,000 dHL-60s to 50 nM fMLP in various concentrations of honey. Cell numbers in top and bottom chambers were measured using a Countess II FL automated cell counter. *∗* indicates a statistically significant difference in cell number from the 0% honey sample. *α*=0.05, measured via one-way ANOVA with Holm-Sidak post hoc.

Figure 4 shows the results of the transwell chemotaxis assay. These results show the total amount of cells, both viable and nonviable, present in the top and bottom wells after the 3-hour incubation period. The significant decrease in migration in the no fMLP control relative to the 0% honey sample indicates that the cells are migrating from the top wells to the bottoms in response to the presence of fMLP throughout the 3-hour experiment. Similarly, the significant decrease in migration in the 0.5%, 3%, and 20% honey samples indicates that Manuka honey reduces this chemotactic response, decreasing the amount of migration to a level at or below the random walk level seen in the no fMLP control. The 20% honey sample had a significant decrease in the number of cells in both the top and bottom chambers, most likely due to the cytotoxicity of this honey concentration and the effect described above with respect to Figure 2.

### 3.5. I*κ*B*α* Phosphorylation

**Figure 5:**
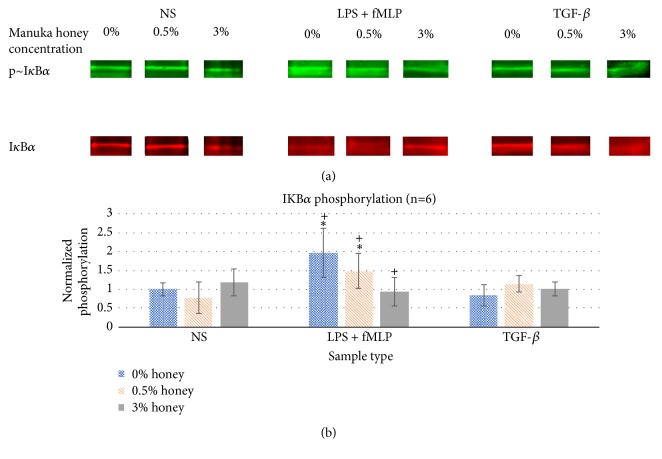
(a) Representative Western blot bands of p~I*κ*B*α* and total I*κ*B*α* for each sample type. (b) I*κ*B*α* phosphorylation expressed as the ratio of p~I*κ*B*α* to total I*κ*B*α*, normalized to NS 0% honey control. *∗* indicates a statistically significant difference from the NS control at that respective honey level. + indicates a statistically significant difference from the other concentrations of honey for that stimulus group. *α*=0.05, measured via two-way ANOVA with Holm-Sidak post hoc.

Figure 5 displays the normalized I*κ*B*α* phosphorylation values for each sample type at 0, 0.5, and 3% honey. As expected, I*κ*B*α* phosphorylation was significantly greater in the LPS + fMLP samples relative to the NS samples in the absence of honey. At 0.5% honey, I*κ*B*α* phosphorylation in the LPS + fMLP group was significantly lowered from the 0% honey samples, but still significantly greater than the NS samples. At 3% honey, I*κ*B*α* phosphorylation in the LPS + fMLP group was significantly lower than both the 0% and 0.5% samples and not significantly different from the NS group. These results indicate that Manuka honey lowers I*κ*B*α* phosphorylation in a dose-dependent fashion when activated via LPS and fMLP, reducing the activity of this inflammatory signal cascade. As expected, TGF-*β* stimulation caused no significant difference in I*κ*B*α* phosphorylation relative to the NS group, and the presence of Manuka honey did not significantly affect I*κ*B*α* phosphorylation in the NS or TGF-*β* group.

## 4. Discussion

The results of this study confirm the hypothesis that Manuka honey reduces neutrophil superoxide release, chemotaxis to fMLP, and the activation of the NF-*κ*B pathway (I*κ*B*α* phosphorylation) when present in concentrations under the cytotoxic limit. The MTS mitochondrial assay and the trypan-exclusion assay establish a cytotoxic limit of Manuka honey beginning between 3-5% v/v and increasing in cell death with increased honey concentration and time (Figures 1-2).  Figure 3(a) indicates that concentrations of 3% honey and above cause an increase in superoxide release during the first hour of culture, suggesting that honey could amplify the initial acute inflammation response. However, Figure 3(c) demonstrates that, after 24 hours of culture, concentrations of 1% honey and above significantly reduce superoxide levels. From Figures 1-2, we know that concentrations of 5% honey and above cause cytotoxicity, which is likely the major contributor to the decrease in superoxide release at these honey levels. Figure 3(c) indicates a significant drop in superoxide release at the 1% honey level, which did not cause any cytotoxicity. Therefore, this drop at 1-3% honey is likely due to honey reducing the superoxide output of the cells via anti-inflammatory effects, rather than cytotoxicity. The chemotaxis and I*κ*B*α* results also indicate that Manuka honey significantly reduces cell migration and I*κ*B*α* phosphorylation in a dose-dependent manner (Figures 4-5). Together, these experimental results indicate that Manuka honey has a significant anti-inflammatory effect on this* in vitro* neutrophil model.

These results agree with findings that have been previously published. Sell et al. conducted cytotoxicity testing using human dermal fibroblasts, human pulmonary microvascular endothelial cells, and human peripheral blood macrophages and observed a cytotoxic limit at 5% v/v Manuka honey and above for all cell types, in line with our results [60]. Leong et al. tested the effect of several varieties of Manuka honeys on human neutrophil superoxide production and found that superoxide inhibition IC_50_ values range from 4.2 to 37.9 mg/mL when stimulated by 0.2 *μ*g/mL phorbol 12-myristate 13-acetate (PMA). As neither the density of their Manuka honey varieties nor the time duration of the assay was reported, a direct comparison between their results and ours cannot be made. Knowing the density would allow the reported weight percent values to be converted to volume percent, enabling a direct comparison of their honey range to ours. However, both sets of results indicate a general trend of honey decreasing superoxide release. The Leong et al. study also involved the topical application of these honey samples to a murine arachidonic acid ear wound model, and they observed decreased neutrophil infiltration into the wound over a 4-hour period after application [4]. This finding concurs with our chemotaxis assay results. A 2018 publication by Gasparrini et al. tested the effect of Manuka honey on NF-*κ*B expression and I*κ*B*α* phosphorylation in LPS-stimulated RAW 264.7 macrophages and found that the honey reduced I*κ*B*α* phosphorylation and NF-*κ*B expression in these cells in a dose-dependent manner [61]. This study agrees with our I*κ*B*α* results, indicating that honey acts through the NF-*κ*B pathway via I*κ*B*α* to reduce inflammatory behavior. Our results in this study thus broadly concur with the results of previously published literature.

The mechanisms through which Manuka honey affects the dHL-60 neutrophil model are unknown and possibly involve a combination of processes initiated by different Manuka honey components. Alvarez-Suarez* et al.* have theorized that polyphenolic components of the honey, such as pinocembrin or pinobanksin, cross the cellular membrane to scavenge intracellular free radicals and trigger 5' AMP-activated protein kinase (AMPK) phosphorylation, increasing antioxidant enzyme expression [10]. Evidence gathered by Gasparrini* et al.* demonstrates that Manuka honey increases the intracellular expression of antioxidant enzymes such as glutathione peroxidase, glutathione reductase, and glutathione s-transferase in macrophages, supporting the Alvarez-Suarez theory [61]. Ultimately, more work is necessary to fully elucidate the mechanisms of action of Manuka honey on neutrophils and other relevant cell types.

Despite the fact that these studies were conducted* in vitro, *certain inferences can be made regarding the role Manuka honey plays in the wound site. When Manuka honey is used clinically as a wound treatment, it is typically either daubed directly onto the wound and covered with a bandage, soaked into a cloth dressing which is then covered with a secondary dry dressing to fasten the honey dressing to the wound, or incorporated into a hydrogel dressing [62]. These methods of application cause the surface of the wound to experience a high concentration of Manuka honey, potentially in excess of the 20% v/v honey concentration used as the highest end of the honey concentration range in this study. Given the cytotoxicity results reported in this study, it is likely that the surface of the wound experiences a “zone of death,” where the bactericidal and osmotic effects combine to kill not only foreign bacteria, but also native human cells, including large numbers of neutrophils which arrive in the wound soon after injury [60]. As the honey diffuses down into the wound environment and becomes more dilute, it is possible that the deeper wound environment encounters honey concentrations closer to the 0.5% and 3% concentrations investigated in this study. The exact concentrations of the honey at relevant penetration depths within the wound are speculative and have yet to be measured* in vivo*. However, according to this study, such honey concentrations would initially promote the neutrophils in the wound to release superoxide, amplifying the acute response within the first hour of application. However, as time continues, this trend reverses, causing the neutrophils to attenuate their superoxide release in the presence of the honey. Meanwhile, the honey's osmotic potential starts a slow net flow of exudate from the deep tissue through the wound bed and out to the wound surface, washing debris and bacteria from the wound site. It is possible that this flow also carries the neutrophils in the wound up to the cytotoxic “zone of death,” and as they are killed, they are initially replaced by naïve neutrophils through the bloodstream. However, as shown by the chemotaxis results in this study, the presence of honey decreases neutrophil chemotaxis in response to the fMLP. Additionally, the I*κ*B*α* phosphorylation results indicate that the honey will begin downregulating the NF-*κ*B pathway, reducing the expression of inflammatory behaviors in neutrophils in the presence of inflammatory stimuli like LPS and fMLP. Thus, in a honey-treated wound, the time of the acute inflammation phase is likely shortened, with fewer and fewer neutrophils chemotaxing to the wound site over time and a reduced inflammation response in the cells encountering inflammatory stimuli.

In addition to their role in wound inflammation, neutrophils also play a part in several inflammation-related pathologies [26, 63]. Excessive neutrophil activity contributes to the formation of atherosclerotic plaque [63], tissue damage associated with chronic obstructive pulmonary disorder [64], tumor formation [65], and inflammatory bowel disease [66–68], among other conditions. The ability of Manuka honey to decrease neutrophil recruitment and inflammatory behavior represents a potential therapeutic opportunity for these pathologies. In particular, a 2008 study by Prakash* et al.* demonstrated that oral administration of Manuka honey significantly reduced colonic inflammation in a rat inflammatory bowel disease model [69]. Although this specific application has yet to be replicated in humans, this study demonstrates the promise of Manuka honey-based therapies in treating inflammation-related pathologies.

The results detailed in this paper demonstrate* in vitro *the specific effects of Manuka honey that enable it to shorten and resolve wound inflammation* in vivo*. They also point towards possible advantages of a lower level, longer term controlled release delivery of the honey to avoid counterproductive cytotoxic effects. As these results show, the inflammation-resolving effects of Manuka honey are present at and below the 3% v/v level. As such, a controlled release of 3% v/v or below Manuka honey from an implanted tissue engineering template would minimize inflammation around the template, allowing faster and more complete healing and tissue-template integration. This effect makes Manuka honey a useful addition to templates for a wide variety of tissue regeneration applications.

Future research will involve examining the effect of Manuka honey on the release of molecular signals (proinflammatory, anti-inflammatory, chemokines, matrix-degrading, and proangiogenic) from this neutrophil model. Additionally, Manuka honey will be incorporated into electrospun tissue templates to examine the effect of honey incorporation on neutrophil NETosis. These experiments will create a better understanding of how honey affects the orchestration of the inflammatory, angiogenic, and inflammation-resolving processes within the wound. As the degree of NETosis has been demonstrated to correlate with fibrous capsule formation and rejection of implanted templates* in vivo*, the NETosis experiments will also inform our understanding of the potential of Manuka honey to improve tissue-template integration and reduce capsule formation. Future research should also include investigation into the effects of other honey varieties on neutrophil inflammatory behaviors. While Manuka honey is the current focus of this paper due to its prevalence in the wound healing field, it is possible that other honey varieties may be as or even more effective at reducing neutrophil inflammatory behaviors. An* in vivo* wound healing model should also be used to confirm the effects described in this paper and measure the honey concentration gradient within the wound.

## 5. Conclusions

In this study, the effect of various concentrations of Manuka honey on NS, LPS + fMLP, and TGF-*β*-stimulated dHL-60 neutrophil models was observed in several ways. First, a moderate cytotoxic effect was found to begin at 3-5% honey and become stronger as honey concentration increased. Concentrations of Manuka honey at 3% and above were found to amplify superoxide production in the first 1-3 hours of culture, but then they suppress superoxide by 24 hours of culture. Furthermore, concentrations of 0.5% and above were found to significantly suppress chemotaxis to fMLP and reduce I*κ*B*α* phosphorylation. These results suggest that Manuka honey has an anti-inflammatory effect on neutrophils, reducing their recruitment to the wound site, their superoxide production, and their intracellular inflammatory signalling.

## Data Availability

The data used to support the findings of this study are included within the supplementary information files.

## Supplementary Materials

Supplementary Materials Supplementary materials are available on the journal website detailing the differentiation of the HL-60 cells and preliminary Western blots used to establish a timeframe for I*κ*B*α* phosphorylation. Additionally, Excel files are provided with the raw data obtained from each experiment described in this paper.
