# Supplementary material for: Manuka Honey Modulates the Inflammatory Behavior of a dHL-60 Neutrophil Model under the Cytotoxic Limit
Source: Int J Biomater. 2019 Feb 25;2019:6132581. doi: 10.1155/2019/6132581 (PMC6415307; doi:10.1155/2019/6132581)
Supplement: Supplementary Materials — are available on the journal website detailing the differentiation of the HL-60 cells and preliminary Western blots used to establish a timeframe for IκBα phosphorylation. Additionally, Excel files are provided with the raw data obtained from each experiment described in this paper. [file 6132581.f1.zip › 6132581.f1/Supplemental Sections 1 and 2 11 25 2018.pdf]

Supplemental Material

1. Quantifying differentiation of Hl-60 cells via morphological changes

The morphology change of HL-60 cells during differentiation is shown in Figure S1A. After treatment with 1.25% v/v DMSO for 6 days, the cell nuclei became more kidney shaped in accordance with the morphological change seen in the literature [42, 48]. DAPI and ActinGreen-stained cells were imaged and classified as either non-differentiated or differentiated. If the cell was elongated and contained a visible divot in its side, or was separated into lobes, it was classified as differentiated; otherwise, it was classified as non-differentiated. As shown in Figure S1B, approximately 69% of the DMSO-treated cells exhibited a change in morphology, while only approximately 9% of non-treated cells exhibited this morphology change. The DMSO treatment thus significantly promoted differentiation to a neutrophil-like phenotype, and these values were comparable to those seen in the literature [34].

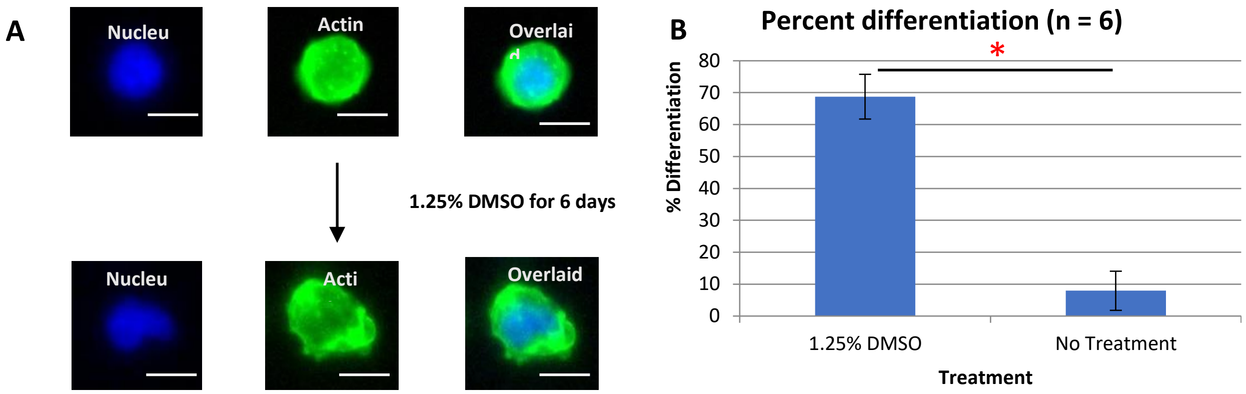

Figure S1. (A) DAPI and ActinGreen-stained HL-60 before and after differentiation with DMSO. Note the change of the nucleus from globular to kidney shaped. Scale bars = 10 µm. (B) Percent differentiation of DMSO treated population, as compared to a non-DMSO-treated population of HL-60s. At least 100 cells of each group were analyzed and counted over 6 images taken from 6 different slides. * indicates statistical significance (α=0.05), two-tailed t-test.

2. Optimizing the IκBα Western blot

To find the time between LPS+fMLP stimulation and maximal IκBα phosphorylation, cell experiments were conducted at various timepoints. These experiments were conducted as described in the methods section, adding the LPS and fMLP at timepoint 0 with no honey, incubating for the specified amount of time, and then placing the well plate on ice, isolating the cytoplasmic proteins, and performing the Western blot as described, running each sample group in 3 Western blot lanes. The first experiment was conducted using an incubation time of 10 minutes, producing the results shown below in Figure S2.

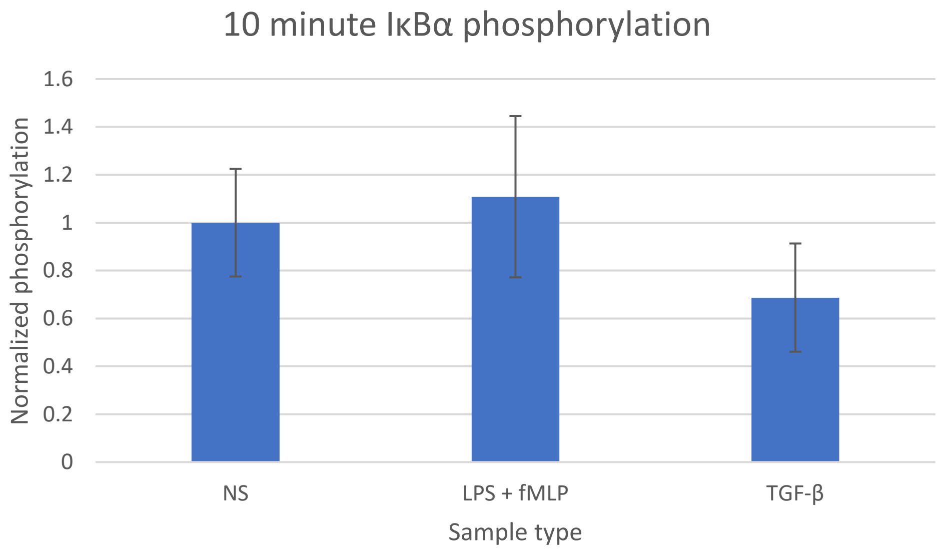

Figure S2. IκBα phosphorylation after 10 minutes of LPS + fMLP expressed as the ratio of phosphorylated IκBα to total IκBα, normalized to the NS samples.

As can be observed in Figure S2, there was no heightened phosphorylation in the LPS + fMLP group at the 10-minute timepoint. Accordingly, the experiment was repeated using a 20 and 30-minute incubation time, producing the results shown in Figure S3.

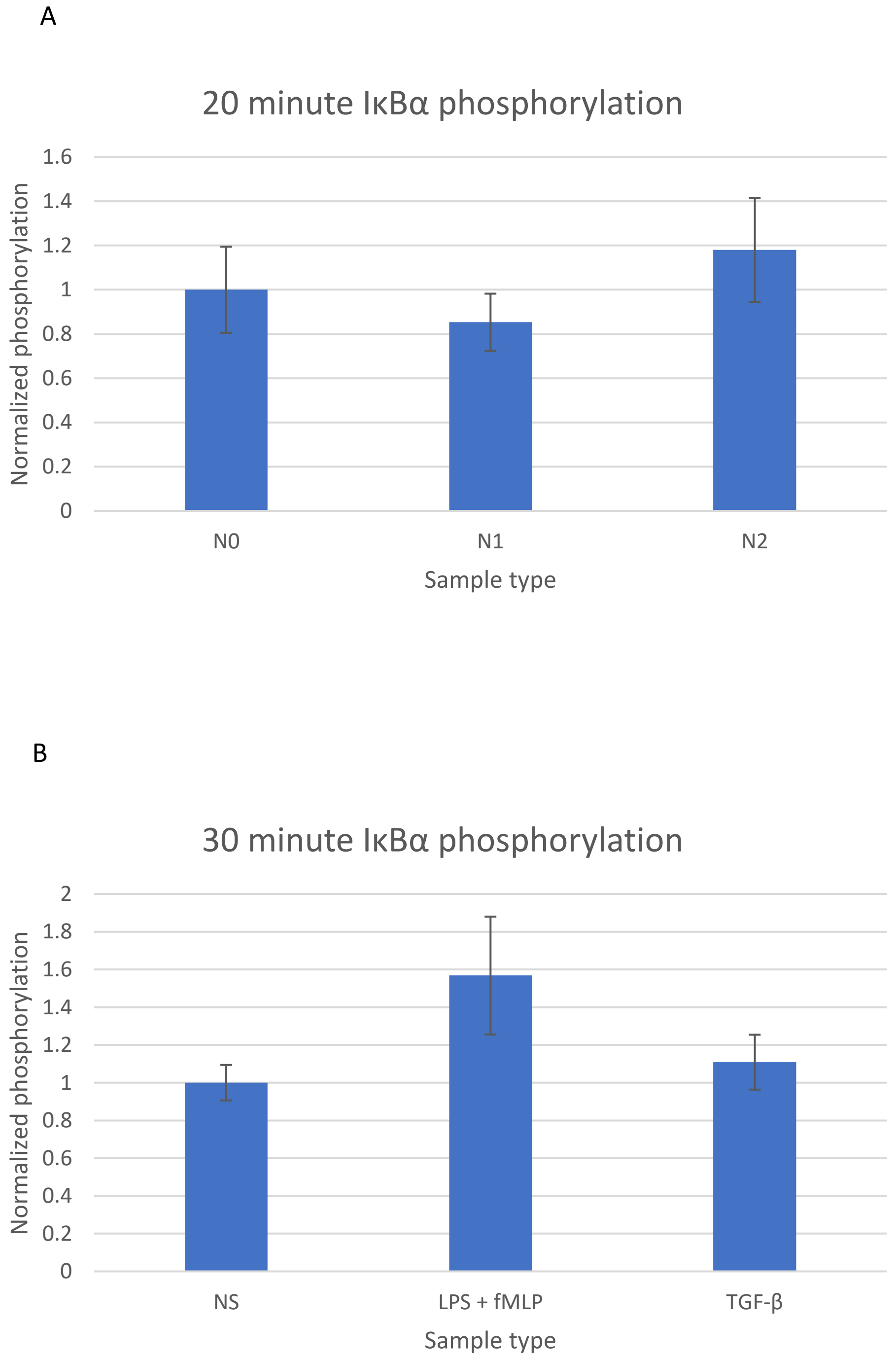

Figure S3. IκBα phosphorylation after 20 (A) and 30 (B) minutes of LPS + fMLP expressed as the ratio of phosphorylated IκBα to total IκBα, normalized to the NS samples.

These results indicated a difference in IκBα phosphorylation between the LPS + fMLP and the NS samples at an incubation time of 30 minutes. In order to determine the point in time of maximum IκBα phosphorylation, a time course experiment was done in which groups of cells were stimulated with LPS +

fMLP and incubated for 31, 32, 33, 34, 35, 36, 37, and 38 minutes. In order to accommodate this greater number of sample groups, each sample type was only run in 1 lane, instead of 3 (n=1). The results of this time course are shown in Figure S4.

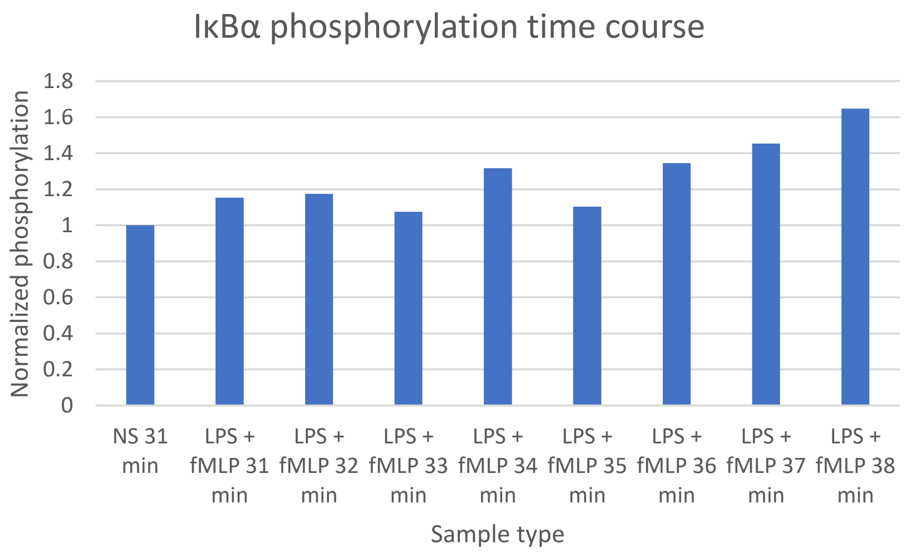

Figure S4. IκBα phosphorylation time course expressed as the ratio of phosphorylated IκBα to total IκBα, normalized to the NS sample. Due to each sample being run only once, there are no standard deviations.

The time course results, shown in Figure S4, indicated that the highest phosphorylation occurred at 38 minutes after LPS + fMLP was added to the culture. Because 38 minutes was the longest timepoint on our range, we did an additional time course experiment using timepoints of 36, 38, 40, and 42 minutes. With 9 wells available on the Western blot gel (the tenth well was used for the protein ladder), we ran two lanes of non-stimulated NS cells incubated for 38 minutes, two lanes of each of the 36, 38, and 40-minute LPS + fMLP samples, and one lane of 42-minute incubated LPS + fMLP samples. The results are shown in Figure S5 below.

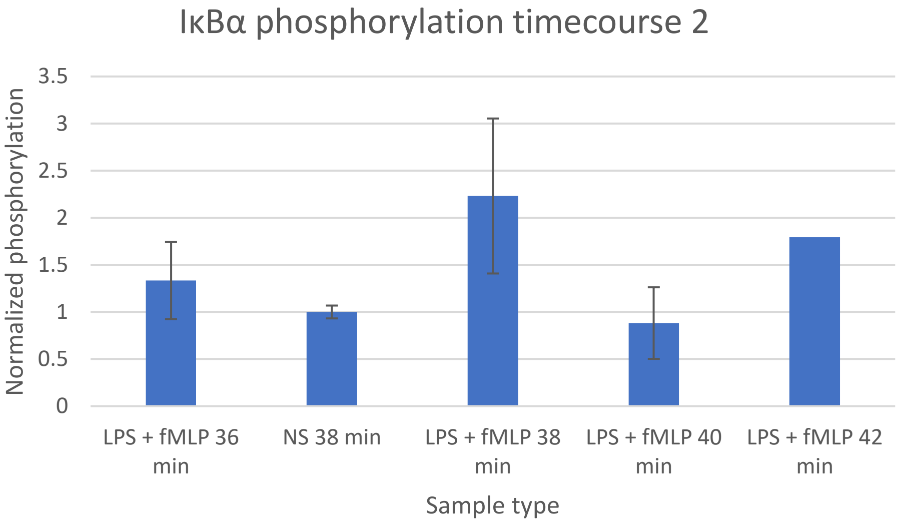

Figure S5. IκBα phosphorylation time course expressed as the ratio of phosphorylated to total IκBα, normalized to the NS sample. Due to the 42-minute sample being run only once, this bar has no standard deviation shown.

As these data indicated that the highest IκBα phosphorylation occurred 38 minutes after the addition of LPS and fMLP to the culture, this timepoint was used to carry out the experiments whose data is shown in Figure 5 of the main text.
