# Supplementary material for: Manuka Honey Modulates the Inflammatory Behavior of a dHL-60 Neutrophil Model under the Cytotoxic Limit
Source: Int J Biomater. 2019 Feb 25;2019:6132581. doi: 10.1155/2019/6132581 (PMC6415307; doi:10.1155/2019/6132581)
Supplement: Supplementary Materials — are available on the journal website detailing the differentiation of the HL-60 cells and preliminary Western blots used to establish a timeframe for IκBα phosphorylation. Additionally, Excel files are provided with the raw data obtained from each experiment described in this paper. [file 6132581.f1.zip › 6132581.f1/Table of contents for raw data files.docx]

Table of contents for raw data files
Article: Manuka Honey Modulates the Inflammatory Behavior of a dHL-60 Neutrophil Model Under the Cytotoxic Limit

Journal: International Journal of Biomaterials
Authors: Benjamin A. Minden-Birkenmaier, Kasyap Cherukuri, Richard A. Smith, Marko Z. Radic, and Gary L. Bowlin^*^

* Correspondence: [glbowlin@memphis.edu](mailto:glbowlin@memphis.edu); Tel.: 901-678-2670

File 1: 1 hour mitochondrial activity data

File 2: 1 hour superoxide results data

File 3: 1 hour tgf beta stimulation superoxide release data

File 4: 3 hour tgf beta stimulation superoxide release data
File 5: 3 hour mitochondrial activity data

File 6: 3 hour mitochondrial activit tgf beta-stimulated data

File 7: 3 hour superoxide results data

File 8: 24 hour mitochondrial activity data tgf b stimulated cells

File 9: 24 hour mitochondrial activity data

File 10: 24 hour tgf beta stimulated cells superoxide release data

File 11: Chemotaxis cell count data

File 12: IKB alpha phosphorylation western blot relative fluorescence

File 13: Raw counts of HL-60 cell morphology under differentiation

File 14: Trypan-exclusion viability assay preliminary experiments and raw data
